# The Bibliometric Analysis of Studies on Physical Literacy for a Healthy Life

**DOI:** 10.3390/ijerph192215211

**Published:** 2022-11-17

**Authors:** María Mendoza-Muñoz, Alejandro Vega-Muñoz, Jorge Carlos-Vivas, Ángel Denche-Zamorano, José Camelo Adsuar, Armando Raimundo, Guido Salazar-Sepúlveda, Nicolás Contreras-Barraza, Nicolás Muñoz-Urtubia

**Affiliations:** 1 Research Group on Physical and Health Literacy and Health-Related Quality of Life (PHYQOL), Faculty of Sport Sciences, University of Extremadura, 10003 Caceres, Spain; 2 Departamento de Desporto e Saúde, Escola de Saúde e Desenvolvimento Humano, Universidade de Évora, 7004-516 Évora, Portugal; 3 Instituto de Investigación y Postgrado, Facultad de Ciencias de la Salud, Universidad Central de Chile, Santiago 8330507, Chile; 4 Public Policy Observatory, Universidad Autónoma de Chile, Santiago 7500912, Chile; 5 Promoting a Healthy Society Research Group (PHeSo), Faculty of Sport Sciences, University of Extremadura, 10003 Caceres, Spain; 6 Comprehensive Health Research Centre (CHRC), University of Évora, 7004-516 Évora, Portugal; 7 Departamento de Ingeniería Industrial, Facultad de Ingeniería, Universidad Católica de la Santísima Concepción, Concepción 4090541, Chile; 8 Facultad de Ingeniería y Negocios, Universidad de Las Américas, Concepción 4090940, Chile; 9 Facultad de Economía y Negocios, Universidad Andres Bello, Viña del Mar 2531015, Chile; 10 International Graduate School, University of Extremadura, 10003 Caceres, Spain

**Keywords:** health, healthy behavior, attitude towards sport, healthy habits, education, children, fitness, well-being, bibliometrics

## Abstract

This article empirically provides a global overview of physical literacy, which allows for the understanding of the structure of the epistemic community studying literacy for healthy living. Publications registered in the Web of Science are analyzed using bibliometrics (spatial, productive, and relational) based on data from 391 records, published between 2007 and April 2022, applying five bibliometric laws and using VOSviewer software for data and metadata processing and visualization. In terms of results, we observe an exponential increase in scientific production in the last decade, with a concentration of scientific discussion on physical literacy in seven journals; a production distributed in 46 countries situated on the five continents, but concentrated in Canada and the United States; co-authored research networks composed of 1256 researchers but with a production concentrated of around 2% of these, and an even smaller number of authors with high production and high impact. Finally, there are four thematic blocks that, although interacting, constitute three specific knowledge production communities that have been delineated over time in relation to health and quality of life, fitness and physical competence, education, and fundamental movement skills.

## 1. Introduction

The theoretical construct of physical literacy (PL) is currently addressed with various meanings in scientific literature [1]. Whitehead [2] highlighted the importance of distinguishing between physical literacy and physical activity; therefore, he offered the definition of physical literacy as “the motivation, confidence, physical competence, knowledge and understanding to value and take responsibility for participation in lifelong physical activity”, positioning it as one of the most widely accepted. Thus, just as reading, writing, listening, and speaking combine to formulate the linguistic literacy that enables a life of reading and communication; PL is a progressive journey in which the different components (physical competence, daily behavior, knowledge and understanding, motivation, and confidence) interact holistically to facilitate a life of participation and enjoyment of physical activity [3].

In this context, PL has been proposed as a key construct for understanding physical activity participation but the lack of an agreed definition and measure has hindered research on the topic [4]. However, it has recently become an important focus of physical education, physical activity, and sports promotion worldwide [5]. This PL construct mainly covers 4 intersection aspects which are presented below: Education, Health, Overweight and Obesity, and Assessment. 

### 1.1. PL and Education

Physical education (PE) has traditionally lacked conceptual cohesion and a shared curricular approach and, therefore, there has always been an absence of a shared philosophy, as different studies reveal [6,7,8]. In this regard, the literature has pointed to PL as the answer [9]. 

Margaret Whitehead [2] stated that PL is not an alternative to PE, nor does it compete with PE. “Physical education” is a subject area in the school curriculum; while “physical literacy” is the goal of PE [2], as several organizations and researchers have previously stated [10,11,12].

PL can be addressed both inside and outside the educational setting. Castelli, Centeio [13] highlight that, within the educational setting, curricula can contribute to PL in different ways: differentiating between structured, unstructured, or informal physical activities (recess), or content-rich physical activity instruction (combining academic concepts with movements). Thus, numerous studies are beginning to address PL both within the PE classroom [14,15] and during out-of-school periods [16,17]. 

### 1.2. PL and Health

Traditionally, there have been more articles attempting to define the concept than articles studying the phenomena, including their possible effect or consequences on other aspects, such as health or well-being [18]. In this respect, Cairney, Dudley [18] conducted one of the first studies to establish a link between PL and health. Specifically, they showed a model that positions PL as a primary determinant of health, through a fully mediated model involving physical activity (PA), positive physiological adaptations resulting from acute and chronic exposures to PA, and possible moderating (interactive) effects of both individual levels, as well as social/environmental conditions that may intervene in the process. In addition, some studies have found relationships between PL and some health-related variables, such as body composition, physical fitness, blood pressure and Health-related quality of life (HRQoL) [19].

### 1.3. PL and Overweight and Obesity

Childhood obesity is one of the major problems and challenges for public health in advanced societies [20] and is closely related to sedentary lifestyles, being considered the 21st-century disease [21,22]. Obesity has been identified as a condition which significantly influences an individual’s level of PL [23]. Thus, understanding how obesity affects childhood PL may help overweight or obese children to live more active lives [24]. In this regard, PL was included in Canada as one of 15 initiatives related to childhood obesity and physical inactivity as early as 2010 [25]. Furthermore, several studies have studied the influence of body composition on it, obtaining direct associations between both parameters [19,24,26].

Therefore, PL could play an important role since a child who has not yet developed a high level of PL will try to avoid physical activity whenever possible, will have minimal confidence in his or her physical ability and will not be motivated to participate in structured physical activity [27]. Thus, the assessment and development of PL may help to explain why children do or do not participate in PA [3], trying to understand how physical activity influences them and may help them to lead a more active life [24]. 

### 1.4. PL Assessment

The growing interest in PL and the benefits that enhancing it can bring have led in recent years to the study of assessment procedures that allow its monitoring and control. Thus, one of the first assessments, the Canadian Assessment of Physical Literacy (CAPL) [28], began to be developed in 2009 because of the persistent demand for objective data on PL. Its goal was to provide a valid, reliable, feasible, and informative tool to help assess the PL of Canadian children. This tool included different domains: fundamental motor skills, physical activity behaviour, physical fitness and knowledge, awareness, and understanding. Currently, this tool has been one of the most widely used tools worldwide, recently used in studies from Denmark [29], China [30], Greece [31], Iran [32], or Spain [26,33]. 

### 1.5. PL Studies

Hence, due to the growing interest in PL, in a recent review carried out in 2021 [34], we can find that there is a multitude of studies that have tried to monitor PL from the different domains that compose it. However, we can only find two explicit PL tools, in addition to the aforementioned CAPL; the Passport for Life (PPL) [35] and PlayFun [36,37] tools. We can also find a study that is under development of a novel tool for this aim, such as The Portuguese physical literacy assessment (PPLA) [38]. Therefore, the growing interest on PL is noteworthy.

Furthermore, the great recent interest in PL monitoring, as highlighted Tremblay and Lloyd [8], may be due to the fact that the results of the assessments can be very useful at different levels. For example, it can help teachers to adapt their planning, head teachers or school leaders to ask for more resources for PL development, and public administrations to convey the importance of PL to policy makers so that they promote and allocate resources for its development [8].

Therefore, due to the current growing interest in the study of PL [1,9], as well as the different fields with which it is being linked, the aim of this study is to assess the development of existing scientific production on PL studies in a comprehensive and up-to-date manner to try to provide an overview to the scientific and practice communities; which is feasible through a bibliometric approach analysing the data and metadata of pre-existing specialised articles.

## 2. Materials and Methods

A set of articles was used as a homogeneous citation base, avoiding the impossibility of comparing indexing databases that use different calculation bases to determine journals’ impact factors and quartiles [39,40,41,42,43], relying on the Web of Science’s (WoS) core collection [44], selecting articles published in journals indexed by the WoS in the Science Citation Index Expanded (WoS-SCIE) and Social Science Citation Index (WoS-SSCI), from a search vector on physical literacy TS = (physical NEAR/0 literacy), with which the query was performed in the WoS Advanced Search module, without restricted temporal parameters, performing the extraction on 21 April 2022. The following types of documents were included: articles, meeting abstracts, reviews, editorial material, book reviews, and letters.

A bibliometric analysis was carried out on a set article obtained for the topic under study. Reviewing the fundamental bibliometric laws:

(1) Exponential science growth or Price’s Law, through the exponential adjustment degree of the annual growth of publications, as a measure of a strong interest among the scientific community to develop studies on physical literacy, conforming a critical researcher mass developing this knowledge topic [45,46].
(2) Publications concentration in journals or Bradford’s Law, distributing the journals in thirds according to the decreasing number of documents published in them, establishing as the nucleus of journals with the highest concentration that covers at least 33% of the total publications [47,48].
(3) Publications concentration in authors or Lotka’s Law, recognizing that in any knowledge field, most of the articles come from a small proportion of prolific authors, who, being identified, can be studied in isolation [49].
(4) Citations concentration in articles or Hirsch index (h-index), thus considering the “n” articles cited at least “n” times or more, a concentration that is extended by transitivity to their authors (author h-index, based on their publications) [50].
(5) Keyword concentration or Zipf’s Law, highlighting the most used keywords in the article set [51].

Finally, VOSviewer software was used to perform the processing and visualization of the dataset, as well as co-occurrence, performing a fragmentation analysis with clustered visualization outputs [52,53].

## 3. Results

**Figure 1:**
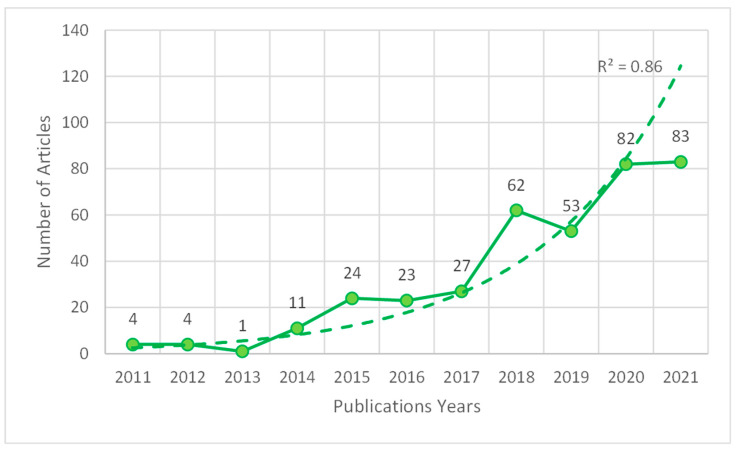
Publications on physical literacy between 2007–2021. solid line: data serie; dashed line: exponential data series trend.

A total of 391 papers were extracted between 2007 and 2022 (open data available in Table S1: PL4HL.xlsx, and Table S2: PL4HL.txt), including current and gap years. However, only between 2011 and 2021 was continuity in publications found. An exponential growth can be seen (R^2^ = 86%), covering a total of 375 articles in that period (Figure 1). 

The resulting document types and extraction databases are detailed in Table 1. Most documents extracted were articles (71.35%), followed by meeting abstracts (14.07%), and the papers principally are published in journals simultaneously indexed in the WoS-SCIE and WoS-SSCI databases (58%).

Bradford’s law was used to identify the key journals that publish on PL [47,48]. According to Bradford’s law, as can be seen in Table 2, seven journals were identified, accounting for 38% of the publications, which can be considered as the core zone of the world scientific discussion on PL. The best-ranked journal according to Bradford’s law is the International Journal of Environmental Research and Public Health (Switzerland) in zone 1, which has published a total of 33 articles. It is also interesting to highlight that 48% of the extracted documents are open access.

**Figure 2:**
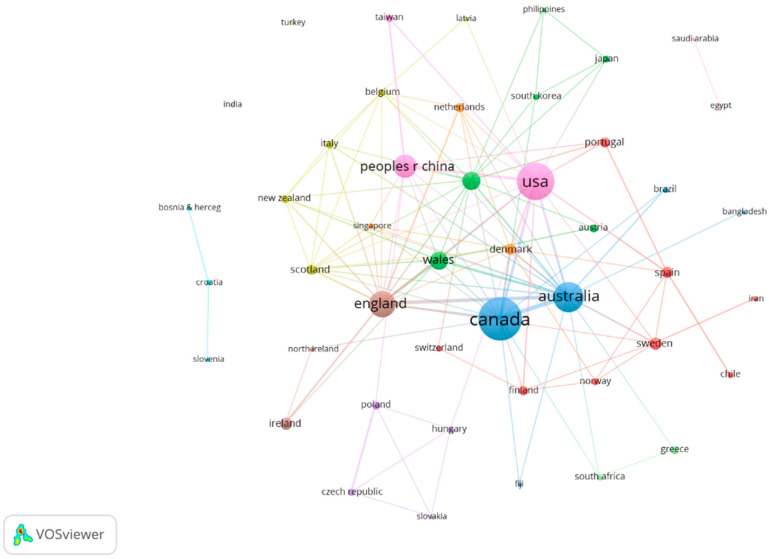
Country/region co-authors graph on physical literacy.

Forty-six countries were found with at least one publication. In terms of co-authorship at the country or regional level, Canada and USA stand out from the rest of the countries (44 countries), with the largest share of knowledge production on physical literacy, including 127 and 92 co-contributions, respectively, by author affiliation (see Figure 2) (attraction: 5; repulsion: −3).

**Figure 3:**
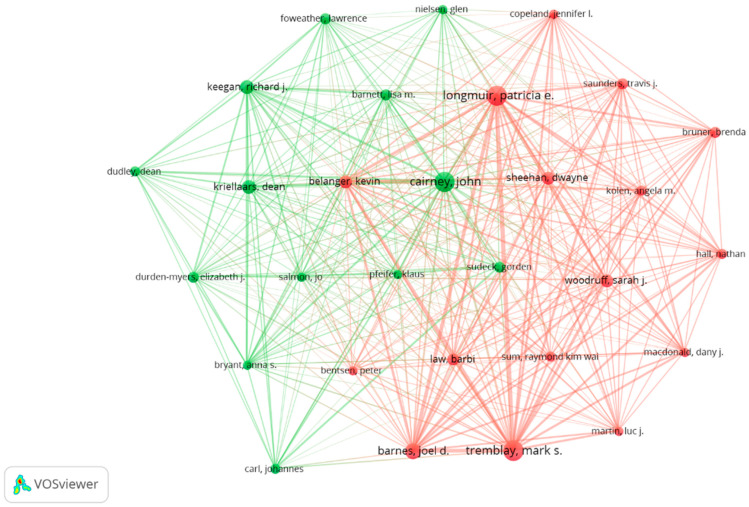
Co-authors graph on physical literacy (only prolific authors).

A total of 391 papers are the result of the scientific production of 1256 authors, so the number of prolific authors is estimated by Lotka’s Law in 35 (Square Root (1256) ≈35.44) [49]. Fifty authors published more than five articles, and 29 researchers released more than six, so a more demanding criterion was taken, a concentration slightly higher than 2% of the world’s authors. When scanning with VOSviewer (Centre for Science and Technology Studies, Leiden University, The Netherlands) the 29 prolific authors with more than six articles, not all are related to each other. Figure 3 shows the cluster plot with a normalisation analysis with the strength of association method (attraction: 10, repulsion: −4) obtained using VOSviewer. 

Table 3 summarises the authors with the number of documents and total citations. In addition, their affiliation and corresponding country are also shown. In this regard and in relation to graph 2, more than half of the prolific authors belong to the country with the highest number of documents, Canada. The USA, the country with the second highest number of documents, does not appear among the affiliations of the prolific authors; however, Australia, the third country with the third highest number of documents, does appear among the affiliations of the prolific authors, with a total of five authors.

**Figure 4:**
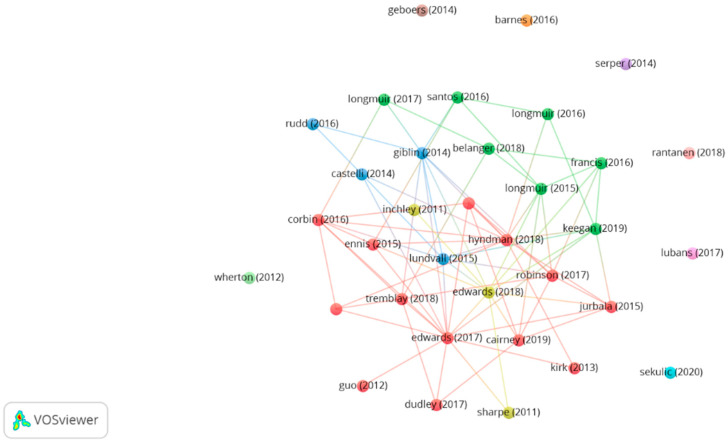
Most cited article graph on physical literacy.

Thirty-four manuscripts were found with 34 or more citations (h-index = 34). Figure 4 displays the cluster plot shown with a strength of association analysis (attraction: 4; repulsion: −4), obtained from VOSviewer. 

Considering prominent authors as those with more than six papers and with at least one of them in h-index 34, the total number of prominent authors drops to 21. The prominent authors, together with the total number of documents, the total number of documents in h-index 34 and the article with the highest number of citations, are shown in Table 4. 

**Figure 5:**
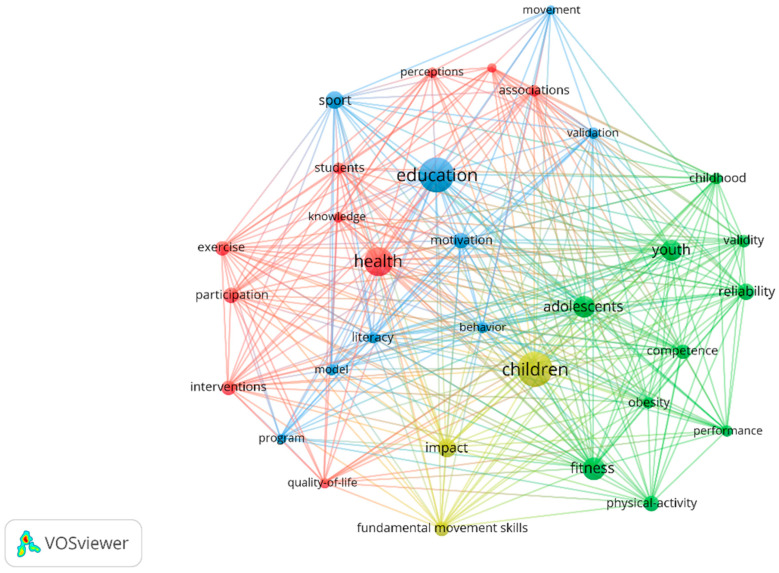
Keywords plus graph.

Concerning the keywords plus, four clusters were identified, as is represented in Figure 5. The first cluster relates health and quality of life with exercise and sedentary behaviour in students. The second cluster is more oriented to fitness and physical competence, relating these to obesity and to parameters such as the reliability and validity of tools in childhood, adolescence, and youth. The third is more oriented towards education, including literacy, motivation, sport, programmes, and validation of tools. Finally, the fourth cluster focuses more specifically on fundamental movement skills and their impact on children. In addition, the most frequently occurring words are education (80), children (79), health (57), and fitness (42). 

## 4. Discussion

Several recent bibliometric studies can be found on PE [54], as well as on its conjunction with other topics, such as sport [55], technology [56], or inclusive education [57]. Bibliometric analyses can also be found on physical activity [58] and its conjunction with themes such as sedentary behaviour and diet [59] or sleep [60]. However, only one study [61] has been found that addresses PL from this perspective. Specifically, this study aimed to map the controversial status of PL and how it is presented by different actors on the academic web. Therefore, there is no study that has conducted a bibliometric analysis of PL.

One of the main findings of this study is the detection of an increasing number of studies between 2011 and 2021, which shows a growing interest of the scientific community in this topic. Most extracted documents were articles (71.35%), followed by meeting abstracts (14.07%), reviews (9.97%), editorial material (2.30%), and other types of documents (2.30%).

Concerning the prolific authors, a mapping in 2021 [61] showed that Tremblay and Longmuir had the highest number of manuscripts (a total of 18), followed by Cairney, Dudley, Sum, Barnes, and Keegan with 10 articles. These outcomes are like those reported in our study, where we observed as Tremblay and Longmuir present the highest number of documents, 25 and 24, respectively, but we found a large increase in the number of Cairney’s documents, with a total of 24. Furthermore, similar to the study by Young, O’Connor [61], we observe that Sum, Barnes, Keegan, and Dudley follow the previous authors (with 12 or more documents), but in addition, they are joined by Kriellaars. These results show the significant growth in the number of papers in just one year for the prolific authors. 

One of the most important themes that can be highlighted from this bibliographic analysis in relation to PL is education. It is one of the words that most frequently appears next to PL, and next to both, motivation, and sport. However, if we analyze the bibliometric analysis on PE by Tomanek and Lis [54], we do not find PL as a relevant keyword, but we find some words related to it such as knowledge, motivation, or competence. Thus, together with the current growing interest in PL, it may lead us to speculate that PL is still a topic to be explored in relation to education. It is supported by the statements by Edwards et al. [1], who highlighted PL as an emerging concept in 2017, in their article entitled “Definitions, foundations and associations of PL: a systematic review. Sports medicine”, which is the most cited article with 139 citations (Table 4).

Another relevant aspect is the growing interest in the study of PL from a health perspective. Proof of this is that the second most cited article (with 100 citations) is “Physical literacy, physical activity and health: Toward an evidence-informed conceptual model” by Cairney et al. [18]. In this sense, the results reported that two of the clusters where the most common keywords were evaluated referred to terms related to this topic, such as health, obesity, or quality of life (Figure 5). Along the same lines, Young, O’Connor [61], in their study, also refer to two clusters closely related to health and highlight that there is a strong positioning in these clusters placing children as the target audience, in line with our results.

Another growing field is the increasing interest of the scientific community in the development of instruments to assess the level of PL in different populations, as can be seen in the results of the present bibliometric analysis, which show that the third most cited article “The Canadian assessment of physical literacy: methods for children in grades 4 to 6 (8 to 12 years)” with a total of 90 citations. Specifically, current research seems to focus its efforts on adapting existing instruments to the own context of each country or region [26,29,30,31,32,33]; and some researchers even focus their research on developing specific tools for their own context [54]. Therefore, the first future research line seems to be directed towards the development of new instruments or the adaptation of previously validated ones; with the aim of adapting them as much as possible to the reality of each country or region and its educational system and customs [33].

Concerning the words class plus it can be observed that in all the clusters the words children, adolescents or young people appear, in addition to education being one of the most relevant. This is consistent with the results of Young et al. [61], who also place ‘physical education’ and ‘children’ among the most relevant keywords. In this sense, all of this leads us to highlight the almost exclusive treatment given to PL from the educational sphere. However, as a limitation, this could be problematic, as the concentration of qualitative research in the school environment directly relates it to the child and adolescent stage [62] and because the concept of PL extends throughout life, it would be necessary as a second future research line to carry out more qualitative research with adults and the elderly in different environments to make the concept more operational throughout life [63]. 

Furthermore, this study has focused on the PL from a generic point of view, for a third future research line on the bibliometric analysis of this term, it would be interesting to specifically address different fields such as health and education, as well as other literacy domains that have been reported in recent publications [64,65], including analyzing more specialized documents, increasing the amount of relevant information on the corresponding topic. Another frequent limitation of bibliometrics is the incompatibility of the various sets of databases in comparative terms, mainly in the comparison for impact due to their different journal, proceeding and book coverage, which forced us to limit ourselves to a specific set of databases (in this case WoS), to perform an analysis on a wider coverage of data fields and metadata [39,40,41,42,43].

## 5. Conclusions

The aim of this study was to assess the development of scientific production on PL. An increasing number of studies was detected between 2011 and 2021, which shows a growing interest of the scientific community in this subject. In terms of reference sources, out of a total of 124, the following seven comprise 38% of the publications: International Journal of Environmental Research and Public Health, BMC Public Health, Journal of Teaching in Physical education, Journal of Sport and Exercise Psychology, Journal of Physical Activity and Health, Research Quarterly for Exercise and Sport, Physical Education, and Sport Pedagogy.

At the author level, out of a total of 1256 authors, only 29 authors were prolific authors with more than six papers. More than half of these authors belong to the country with the highest number of papers, Canada (32.48%). In addition, 21 of the authors were considered prominent, considering those with more than six papers and with at least one of them in the h-index 34.

The set of articles concentrates the topics in four clusters, one related to health, one to fitness and obesity, one to education and, finally, one to fundamental movement skills. In addition, the most used keywords were education (80), children (79), health (57), and fitness (42).

## Figures and Tables

**Table 1:** Extracted document types by WoS index databases.

Document Type	WoS-SCIE; WoS-SSCI	WoS-SSCI	WoS-SCIE	WoS-SCIE; WoS-CPCI-S *	WoS-SSCI; WoS-CPCI-SSH **	Total
Article	158	59	44	0	0	261
Meeting Abstract	25	8	3	13	6	55
Review	27	8	2	0	0	37
Article; Early Access	7	11	0	0	0	18
Editorial Material	6	1	2	0	0	9
Correction	0	1	2	0	0	3
Letter	1	1	1	0	0	3
Book Review	1	1	0	0	0	2
Review; Early Access	2	0	0	0	0	2
Biographical-Item	0	1	0	0	0	1
Total	227	91	54	13	6	391
Percentage (%)	58%	23%	14%	3%	2%	100%

* Conference Proceedings Citation Index—Science. ** Conference Proceedings Citation Index—Social Science and Humanities.

**Table 2:** Journals with more than 4 publications ranked according to Bradford’s Law.

Source	Total Documents	Total Citations *	Quartile (JCR2021)	Cumulative Frequency	Open Access
International Journal of Environmental Research and Public Health	33	136	1	8%	100%
BMC Public Health	30	506	2	16%	100%
Journal of Teaching in Physical education	26	415	1	22%	50%
Journal of Sport and Exercise Psychology	19	15	2	27%	5.3%
Journal of Physical Activity and Health	16	179	2	31%	12.5%
Research Quarterly for Exercise and Sport	14	151	2	35%	7.1%
Physical Education and Sport Pedagogy	14	65	1	38%	28.6%
Sources (102) outside the zone nucleus	239	2806	N/A	100%	43.5%
Total	391	4273	N/A	100%	48%

N/A: not applicable, * Times Cited, WoS Core.

**Table 3:** Prolific authors with affiliation, country, total number of documents, and total number of citations.

Prolific Authors	Affiliation	Country	Documents	Citations
Tremblay, Mark S.	Children’s Hospital Eastern Ontario University of Ottawa	Canada	25	576
Cairney, John	The University of Queensland University of Toronto	Australia Canada	24	347
Longmuir, Patricia E.	Children’s Hospital Eastern Ontario University of Ottawa	Canada	24	464
Sum, Raymond Kim Wai	The Chinese University of Hong Kong	China	19	119
Barnes, Joel D.	Children’s Hospital Eastern Ontario	Canada	16	316
Kriellaars, Dean	University of Manitoba	Canada	13	256
Keegan, Richard J.	University of Canberra	Australia	12	356
Dudley, Dean	Macquarie University	Australia	12	206
Belanger, Kevin	Children’s Hospital Eastern Ontario	Canada	11	168
Sheehan, Dwayne	Mount Royal University’s	Canada	11	128
Woodruff, Sarah J.	University of Windsor	Canada	11	168
Law, Barbi	Nipissing University	Canada	10	101
Barnett, Lisa M.	Deakin University	Australia	9	62
Bruner, Brenda	Nipissing University	Canada	9	101
Durden-Myers, Elizabeth J.	Liverpool John Moores University	England	8	134
Foweather, Lawrence	Liverpool John Moores University	England	8	60
Saunders, Travis J.	University of Prince Edward Island	Canada	8	157
Sudeck, Gorden	University of Tübingen	Germany	8	115
Carl, Johannes	Friedrich-Alexander-Universität Erlangen-Nürnberg	Germany	7	34
Hall, Nathan	University of Winnipeg	Canada	7	101
Kolen, Angela M.	St. Francis Xavier University	Canada	7	95
Bryant, Anna S.	Cardiff Metropolitan University	Wales	6	279
Copeland, Jennifer L.	University of Lethbridge	Canada	6	94
Macdonald, Dany J.	University of Prince Edward Island	Canada	6	101
Martin, Luc J.	Queen’s University	Canada	6	94
Pfeifer, Klaus	Friedrich-Alexander-Universität Erlangen-Nürnberg	Germany	6	34
Salmon, Jo	Deakin University	Australia	6	57

**Table 4:** Prominent authors with at least one documents in h-index 34, their most cited paper and citations from those documents.

Author	Total Documents	Documents in h-Index 34	Most Cited Document	Times Cited *
Tremblay, Mark S.	25	7	Longmuir, P.E., Boyer, C., Lloyd, M., Yang, Y., Boiarskaia, E., Zhu, W., and Tremblay, M.S. (2015). The Canadian assessment of physical literacy: methods for children in grades 4 to 6 (8 to 12 years). *BMC public health*, *15*(1), 1–11.	90
Longmuir, Patricia E.	24	6		
Cairney, John	24	3	Cairney, J., Dudley, D., Kwan, M., Bulten, R., and Kriellaars, D. (2019). Physical literacy, physical activity and health: Toward an evidence-informed conceptual model. *Sports Medicine*, *49*(3), 371–383.	100
Kriellaars, Dean	13	2		
Dudley, Dean	12	3		
Barnes, Joel D.	16	4	Francis, C.E., Longmuir, P.E., Boyer, C., Andersen, L.B., Barnes, J.D., Boiarskaia, E., ..., and Tremblay, M.S. (2016). The Canadian assessment of physical literacy: development of a model of children’s capacity for a healthy, active lifestyle through a Delphi process. *Journal of Physical Activity and Health*, *13*(2), 214–222.	55
Keegan, Richard J.	12	3	Edwards, L.C., Bryant, A.S., Keegan, R.J., Morgan, K., and Jones, A.M. (2017). Definitions, foundations and associations of physical literacy: a systematic review. *Sports medicine*, *47*(1), 113–126.	139
Bryant, Anna S.	6	3		
Belanger, Kevin	11	1	Belanger, K., Barnes, J.D., Longmuir, P.E., Anderson, K.D., Bruner, B., Copeland, J.L., ..., and Tremblay, M.S. (2018). The relationship between physical literacy scores and adherence to Canadian physical activity and sedentary behaviour guidelines. *BMC Public Health*, *18*(2), 1–9.	35
Sheehan, Dwayne	11	1		
Woodruff, Sarah J.	11	1		
Law, Barbi	10	1		
Bruner, Brenda	9	1		
Hall, Nathan	7	1		
Kolen, Angela M.	7	1		
Copeland, Jennifer L.	6	1		
Macdonald, Dany J.	6	1		
Martin, Luc J.	6	1		
Barnett, Lisa M.	9	2	Rudd, J., Butson, M.L., Barnett, L., Farrow, D., Berry, J., Borkoles, E., and Polman, R. (2016). A holistic measurement model of movement competency in children. *Journal of sports sciences*, *34*(5), 477–485.	47
Saunders, Travis J.	8	2	Longmuir, P.E., Boyer, C., Lloyd, M., Borghese, M.M., Knight, E., Saunders, T.J., ..., and Tremblay, M.S. (2017). Canadian Agility and Movement Skill Assessment (CAMSA): Validity, objectivity, and reliability evidence for children 8–12 years of age. *Journal of sport and health science*, *6*(2), 231–240.	56
Salmon, Jo	6	1	Keegan, R.J., Barnett, L.M., Dudley, D.A., Telford, R.D., Lubans, D.R., Bryant, A.S., ..., and Evans, J.R. (2019). Defining physical literacy for application in Australia: A modified delphi method. *Journal of Teaching in Physical Education*, *38*(2), 105–118.	36

* Times Cited according to WoS Core Collection (until 21 April 2022).

## Data Availability

The analyzed dataset has been included as Supplementary Materials.

## Supplementary Materials

The following supporting information can be downloaded at: https://www.mdpi.com/article/10.3390/ijerph192215211/s1, Table S1: PL4HL.xlsx (for MS-Office), Table S2: PL4HL.txt (for VOSviewer).
